# In Vitro Studies Demonstrate Antitumor Activity of Vanadium Ions from a CaO-P_2_O_5_-CaF_2_:V_2_O_5_ Glass System in Human Cancer Cell Lines A375, A2780, and Caco-2

**DOI:** 10.3390/ijms24021149

**Published:** 2023-01-06

**Authors:** Cristian Lujerdean, Marius Zăhan, Daniel Severus Dezmirean, Răzvan Ștefan, Dorina Simedru, Grigore Damian, Nicoleta Simona Vedeanu

**Affiliations:** 1 Faculty of Animal Science and Biotechnology, University of Agricultural Sciences and Veterinary Medicine Cluj-Napoca, 400372 Cluj-Napoca, Romania; 2 INCDO-INOE 2000, Research Institute for Analytical Instrumentation Subsidiary (ICIA) Cluj-Napoca, 67 Donath Street, 400293 Cluj-Napoca, Romania; 3 Faculty of Physics, Babes-Bolyai University, 400084 Cluj-Napoca, Romania; 4 Department of Pharmaceutical Physics-Biophysics, Faculty of Pharmacy, “Iuliu Hatieganu” University of Medicine and Pharmacy, Pasteur 6, 400349 Cluj-Napoca, Romania

**Keywords:** phosphate glasses, V_2_O_5_ ions, pH measurements, Fourier transform infrared (FT-IR), antitumor activity, MTT assay

## Abstract

In this research, we investigated the structural and biological properties of phosphate glasses (PGs) after the addition of V_2_O_5_. A xV_2_O_5_∙(100 − x)[CaF_2_∙3P_2_O_5_∙CaO] glass system with 0 ≤ x ≤ 16 mol% was synthesized via a conventional melt-quenching technique. Several analysis techniques (dissolution tests, pH, SEM-EDS, FT-IR, and EPR) were used to obtain new experimental data regarding the structural behavior of the system. In vitro tests were conducted to assess the antitumor character of V_2_O_5_-doped glass (x = 16 mol%) compared to the matrix (x = 0 mol%) and control (CTRL-) using several tumoral cell lines (A375, A2780, and Caco-2). The characterization of PGs showed an overall dissolution rate of over 90% for all vitreous samples (M and V1–V7) and the high reactivity of this system. EPR revealed a well-resolved hyperfine structure (hfs) typical of vanadyl ions in a C*_4v_* symmetry. FT-IR spectra showed the presence of all structural units expected for P_2_O_5_, as well as very clear depolymerization of the vitreous network induced by V_2_O_5_. The MTT assay indicated that the viability of tumor cells treated with V7-glass extract was reduced to 50% when the highest concentration was used (10 µg/mL) compared to the matrix treatment (which showed no cytotoxic effect at any concentration). Moreover, the matrix treatment (without V_2_O_5_) provided an optimal environment for tumor cell attachment and proliferation. In conclusion, the two types of treatment investigated herein were proven to be very different from a statistical point of view (*p* < 0.01), and the in vitro studies clearly underline the cytotoxic potential of vanadium ions from phosphate glass (V7) as an antitumor agent.

## 1. Introduction

One of the most promising research directions for compounds containing vanadium is their potential anticancer activity [1]. According to the World Health Organization (WHO), in 2020, there were 19.3 million cases of cancer and almost 10.0 million deaths worldwide, which increased the burden on medical systems all over the world [2]. Moreover, cancer is a condition that can develop from almost any cell and therefore anywhere in the body, and it continues to be a leading cause of death worldwide. Cancers that most often affect women include colorectal, skin, and ovarian cancers [3]. 

Vitreous materials have numerous possible compositions and a wide range of applications [4,5,6]. Among these, a special class of glasses is phosphate glasses (PGs). PGs have been developed and intensively studied, mainly in the biomedical field (such as for surgical implants used in tissue engineering, wound dressings composed of bioactive glass microfibers for wound healing and the promotion of angiogenesis, bioactive glass-based composite biomaterials, drug delivery through antibacterial ions (silver and copper ions), and dental implants) [1,4,7,8,9] and in technological applications (such as IC packaging, thick-film technology, glass–metal sealing, flat-panel display sealing, as a host matrix for the vitrification of radioactive waste, and as a host material for lasers) [10,11,12,13]. The properties that make PGs promising candidates in biomedical applications are closely related to their molecular structure, which is generally described using the Q^n^ formalism, where *n* represents the number of bridging oxygen atoms in each tetrahedron [12,13]. Their greatest advantage is their solubility in aqueous solutions and the possibility of being tuned over many orders of magnitude by tailoring the glass composition [14,15]. Another benefit from a biomedical standpoint is the fact that these glasses can be doped with ions routinely found inside the body. Once dissolved in a biological fluid, they can initiate a wide range of responses by releasing ions into the local environment [15].

Many PGs are materials with excellent bioresorbability [16,17] and very high biocompatibility with living tissues [11,18], offering great potential for tissue engineering applications. 

The addition of transition metal oxides (TMOs) in the composition of phosphate glass typically leads to the depolymerization of the vitreous phosphate network and to the formation of new P–O–TM bonds [13]. These structural modifications are linked to the amount of doping and have a strong influence on the physical, chemical, and magnetic properties of these glasses [19,20,21,22]. Moreover, including TMOs in the P_2_O_5_ network not only improves their properties but can also have beneficial effects from a biological perspective [23].

Vanadium (V) is one of the most suitable transition metals that can be incorporated into PGs, as it is characterized by a partially filled *3d* layer and can exist in at least two valence states in these glasses [24]. These valence states are influenced by synthesis conditions, the raw chemicals used, and the total amount of V_2_O_5_ in the glass [25].

In recent years, numerous studies have reported antitumoral activity for many vanadium-containing compounds [26]. Thus, vanadium compounds exert their antitumor effects through the following molecular and cellular mechanisms:

(i) Fragmentation activities that directly target DNA. Studies conducted by Mohamadi et al. [27] through cyclic voltammetry, competitive fluorescence assays, and electronic absorption spectroscopy showed a groove binding of a complex containing vanadium to salmon sperm DNA, which was also accompanied by a partial insertion of the ligand between the base stacks of the DNA. Furthermore, this mononuclear diketone-based oxido vanadium (IV) complex showed significant cytotoxicity against multiple cancer cell lines (i.e., breast, liver and colon).
(ii) Disruption of the redox balance due to an increase in reactive oxygen species (ROS). Reactive oxygen species are well known to promote a multitude of aspects in tumor development and progression and are detected in all cancers cells. Vanadium salts such as sodium metavanadate (NaVO_3_) or vanadium (IV) sulfate oxide (VOSO_4_) have been reported to induce a significant increase in ROS level in human lung cancer cells [28,29]. Furthermore, a polyacrylate derivative of peroxovanadate was shown to inhibit the growth of lung carcinoma cells [30]. Finally, a vanadium dioxide nanocoating (VO_2_-modified) quartz surface prepared by Li et al. [31] increased intracellular ROS levels in cholangiocarcinoma cells.
(iii) Apoptosis or programmed cell death. Apoptosis serves the purpose of removing DNA-damaged cells that could lead to carcinogenesis. This process is disturbed in cancer cells, which have a mechanism of avoiding it. The literature reports that this mechanism was induced in an oral squamous cell carcinoma cell line [32] and in human anaplastic thyroid carcinoma cells [33] by sodium orthovanadate (Na_3_VO_4_). In gastric cancer lines, a vanadium complex used by Wang et al. [34] also induced apoptosis. Furthermore, in human breast cancer cell lines, this effect was obtained through a vanadium complex combined with the flavonoid quercetin [35].
(iv) Cell cycle arrest. It is already well known that cancer cells are characterized by a dysregulation of the cell cycle, resulting in aberrant cell proliferation and their high genetic instability [36]. Sodium metavanadate was reported to cause G2/M cell cycle arrest in prostate cancer cells in a study conducted by Liu et al. [37]. A similar G2/M cell cycle arrest mechanism was described for the effect of sodium metavanadate on papillary thyroid carcinoma-derived cells [38], for an oxido vanadium complex containing a quinolinium cation in pancreatic cancer cell lines [26], and for an oxido vanadium complex with phenanthroline in hepatocellular carcinoma cell lines [39]. All these antitumoral effects are mediated by the cyclin/CDK complexes, which play a key role in checkpoints of the cell cycle.

Against this background, in the present study, we evaluated the effects of vanadium on the structural and biological properties of a xV_2_O_5_∙(100 − x) [CaF_2_∙3P_2_O_5_∙CaO] glass system with 0 ≤ x ≤ 16 mol%. The structural properties of the glass system were examined by a variety of techniques, namely electron paramagnetic resonance (EPR), Fourier transform infrared spectroscopy (FT-IR), and scanning electron microscopy with energy-dispersive spectroscopy (SEM-EDS). pH measurements and dissolution tests were used to evaluate both the release behavior and bioactivity of this glass system. Furthermore, in order to explore and quantify the antitumoral character of vanadium ions from the phosphate glass, we used in vitro biological tests and a tetrazolium-based MTT assay, respectively, on three different human cancer cell lines (A375, A2780, and Caco-2).

## 2. Results and Discussion

### 2.1. Structural Characterization

#### 2.1.1. Dissolution Tests

Pure or high-phosphorus-content glasses are characterized by relatively low chemical durability compared to silicate- and borate-based glasses [40].

According to the literature, the dissolution of PGs in aqueous media involves two pivotal stages, namely hydration (softening stage) and hydrolysis (breaking stage) [41].

As a result of the hydration reaction, P–O–P bonds in the hydrated layer break, and a hydrolysis reaction takes place (second stage). This stage is a process of degradation of the glass network (from outside to inside) and is highly dependent on ambient pH [40].

**Figure 1:**
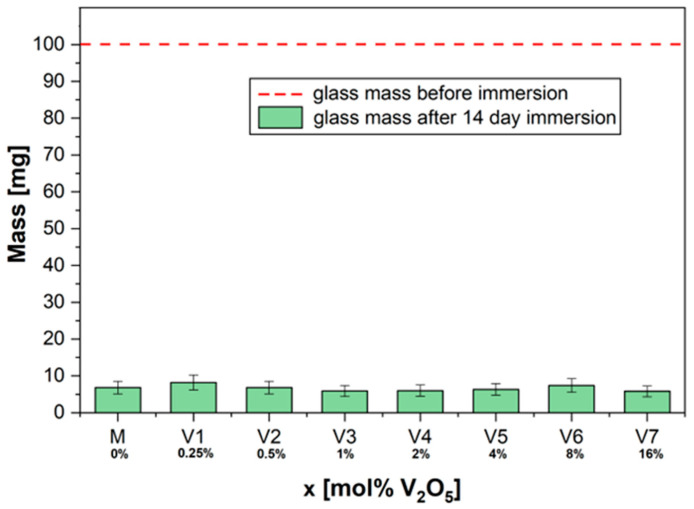
The solubility of glass powders: dry mass before and after immersion for 14 days in 10 mL deionized water.

In our case, the solubility of the glasses with xV_2_O_5_∙(100 − x)[CaF_2_∙3P_2_O_5_∙CaO], where x = 0.25, 0.5, 1, 2, 4, 8, and 16 mol%, was increased for all compositions (Figure 1). The doping (V_2_O_5_) of the structure of phosphate glasses did not affect their dissolution rate. After 14 days of incubation, the glass samples showed a mass loss of over 90% of their initial mass.

No considerable change in chemical durability was observed for the sample with the highest doping concentration (x = 16 mol%).

Bunker et al. [42] developed a general description of the dissolution reactions between PGs and water. In our case, water hydrated the phosphate anions by reacting with the metal V–O–P bonds that link neighboring P anions according to the following equation:

─P─ O^−^ (Ca^2+^; V^n^) O^−^ ─ P + 2H_2_O → ─ P─OH HO─P─ + (Ca^2+^; V^n^) (OH)_2_
 |            | (F^−^)            (F^−^)
n = oxidation states (2+, 3+, 4+ or 5+)                (1)

The results presented here can be explained by the action of water molecules (H^+^, H_3_O^+^) that diffuse into the surface structure and break the weakest P–O–P or P–O–V chains. The size and the shape of the glass, as well as the glass surface area, play a dominant role in ion exchange; this corrosion property is determined by the ratio between the area and the volume of the glass [41]. The dissolution/corrosion behavior of glasses with the CaO-P_2_O_5_-CaF_2_:V_2_O_5_ depends on the network strength, which is based mainly on the glass composition.

#### 2.1.2. pH Evolution

**Figure 2:**
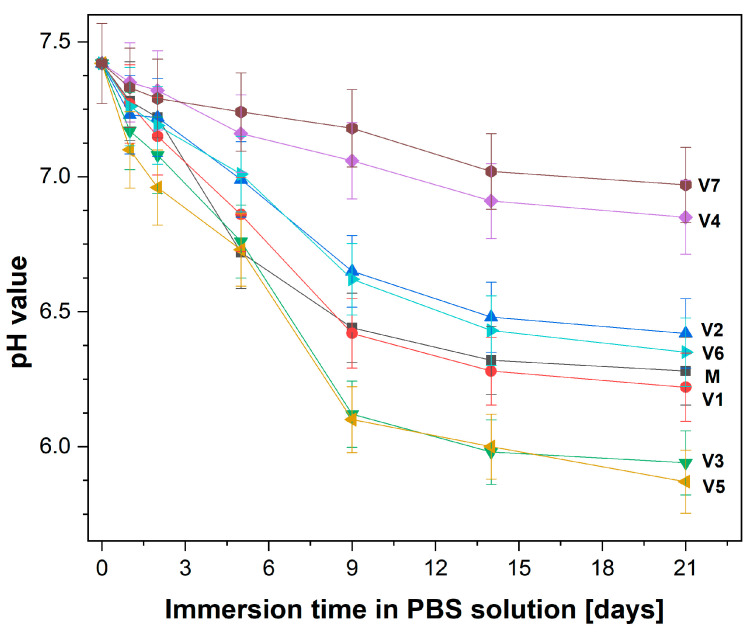
pH evolution of phosphate-buffered saline (PBS) solution as a result of glass sample immersion over 21 days at 37 °C.

During 21 days of immersion, the evolution of pH values in a phosphate buffer solution (PBS) was measured at different time intervals; the results are illustrated in Figure 2. All investigated compositions caused a decrease in the pH of PBS. A significantly strong decrease was visible on the first day. Then, a slower decrease over the rest of the incubation time was noticed. According to the procedure, several measurements of pH were performed, with the most important control points being: on day 0, an initial pH value of 7.43; on day 9 pH values ranging from 7.18 to 6.10; and on day 21, pH values ranging from 6.97 to 5.94.

According to the literature, the pH values produced by the presence of PGs are a consequence of their dissolution, which generates some acidic species as a result of an ion exchange process [42,43,44].

In our research, the pH gradually decreased as immersion time increased. This might be due to the formation of phosphoric acid in relation to H_2_PO_4_^−^ entities [44]. Therefore, phosphate ions at the glass surface are released into the PBS solution through the breakage of P−O−P/P−O−V bonds associated with the PO_4_ units.

H_3_PO_4_ + H_2_O ↔ H_2_PO_4_^−^ + H_3_O^+^ (K_1_ = 7.1 × 10^−3^)

H_2_PO_4_^−^ + H_2_O ↔ HPO_4_^2−^ + H_3_O^+^ (K_2_ = 6.3 × 10^−8^)

HPO_4_^2−^ + H_2_O ↔ PO_4_^3−^ + H_3_O^+^ (K_3_ = 4.4 × 10^−13^)

Additionally, the fluorine content can have a notable impact on the pH values due to the ion exchange process [45].

Similarly, just as Ca^2+^ ions on the glass surface combine with the PBS solution in exchange for H^+^ ions from the solution (from the dissociation of water into H^+^ and OH^−^) [46], F^−^ ions are exchanged for OH^−^ groups. Thus, hydroxyl is removed from the solution, and a weak acid–hydrofluoric acid is formed. If the fluoride content increases, more hydrofluoric acid is formed, and the pH increase is less noticeable [47].

#### 2.1.3. SEM-EDS

**Figure 3:**
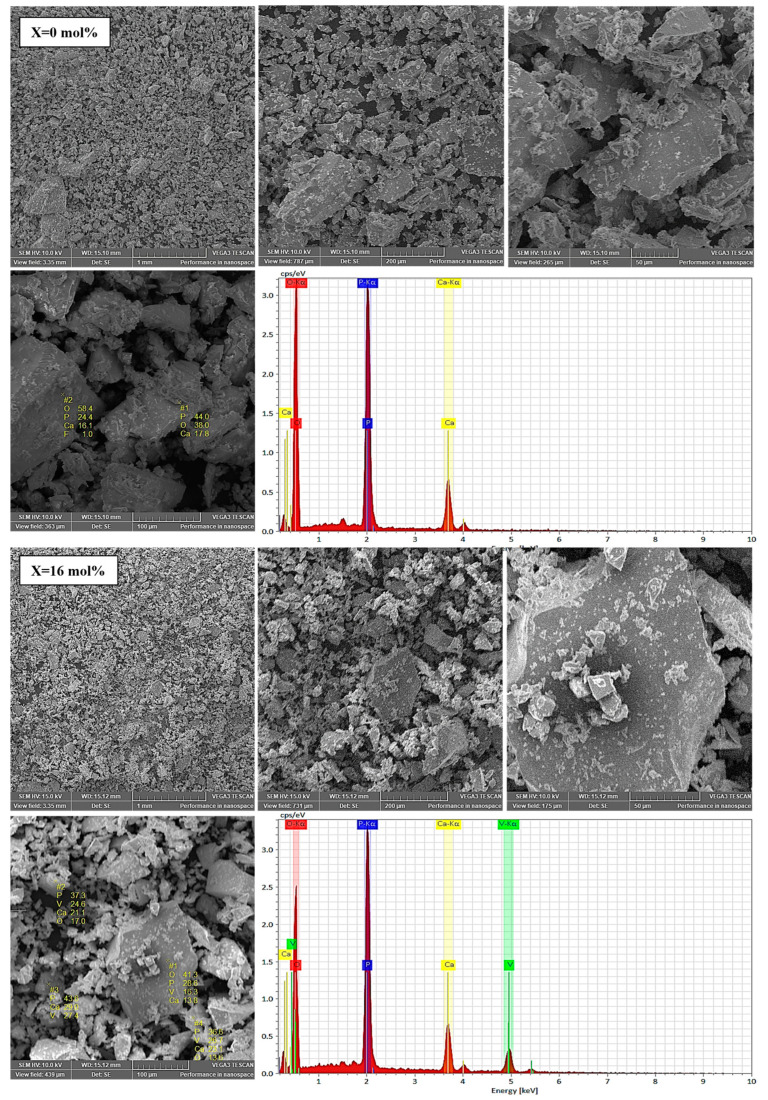
SEM micrographs and EDS analysis for glass samples with x = 0 and 16 mol% V_2_O_5_ used in the dissolution experiments. Colour legend: O (red), Ca (yellow), V (green) and P (blue).

The surface morphologies of two relevant samples (M and V7) are shown in Figure 3. SEM analyses showed that all glasses have numerous microparticles with irregular shapes and a wide distribution of sizes. No spherical particles were observed.

The presence of vanadium was confirmed by EDS analysis in all studied phosphate glasses, except for the host glass (matrix), which was free of doping. In addition, all constituent elements (P, O, Ca, and V) were confirmed in quantities analogous to the initial nominal composition (Table 1).

A decrease in both oxygen and calcium occurred in samples V1 to V7 as the doping concentration increased, whereas the amount of phosphorus remained relatively unchanged, with range of values between 28.50–33.49 wt%.

**Figure 4:**
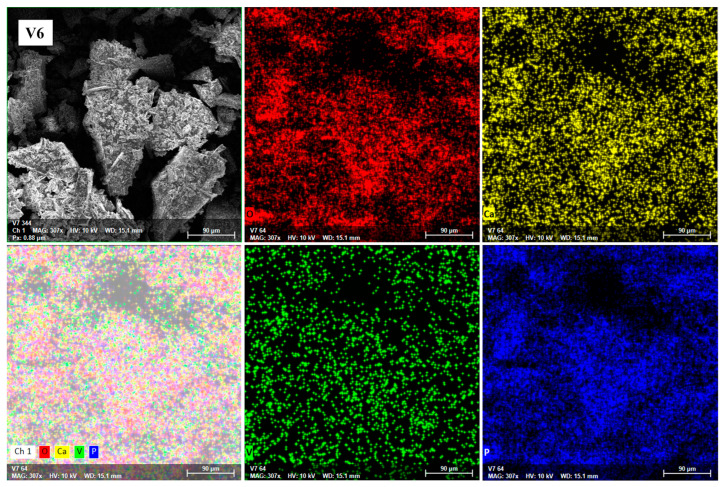
EDS mapping of O (red), Ca (yellow), V (green), and P (blue) of glass powder with x = 8 mol% V_2_O_5_ before the dissolution experiments.

To confirm the homogeneous structure of the studied glasses, SEM characterizations combined with EDS were performed. EDS mapping analysis was performed for the entire glass system. A representative image of V6 glass is shown in Figure 4; the red, blue, yellow, and green dots correspond to O, P, Ca, and V distribution states, respectively.

Our data show a good chemical homogeneity of the detected elements in all glasses (M and V1–V7). Moreover, the results of the elemental analysis are in accordance with the compositional elements used in their preparation, supporting and confirming the existence of strict stoichiometric ratios among the constituent atoms.

#### 2.1.4. EPR

**Figure 5:**
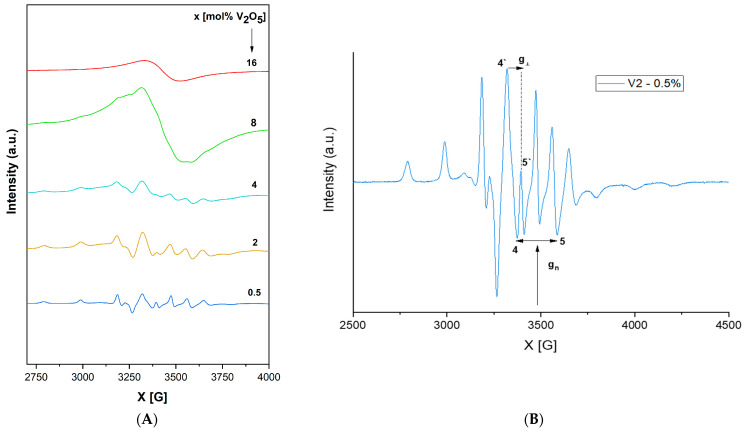
(**A**). EPR spectra of a xV_2_O_5_∙(100 − x) [CaF_2_∙3P_2_O_5_∙CaO] glass system with 0 ≤ x ≤ 16 mol%. (**B**). Individual EPR spectra of a xV_2_O_5_∙(100 − x) [CaF_2_∙3P_2_O_5_∙CaO] glass system with x = 0. 5 mol%.

The EPR spectra of a vanadium-doped xV_2_O_5_∙(100 − x) [CaF_2_∙3P_2_O_5_∙CaO] glass system with 0 ≤ x ≤ 16 mol% are presented in Figure 5A.

According to our previous research on V_2_O_5_-doped phosphate glasses [48,49,50], vanadium oxide acts both as a network modifier for low- and medium-vanadium-content glasses and a network former for higher-content glasses (due to the similarity of VO_4_ tetrahedral units to phosphate units).

Vanadium has five valence electrons that can be lost and in glasses or glass ceramics and can exist in different ionic forms (i.e., V^3+^, V^4+^, and V^5+^) [49,50,51].

For the glass system investigated in the present study, as shown in Figure 5A,B, EPR spectra show a well-resolved *hfs* (hyperfine structure) for 0.25 ≤ x ≤ 4 mol %, with a structure based on 16 lines (8 in the parallel and 8 in the perpendicular band) attributed to the interaction of a 3d^1^ spin electron with ^51^V nuclear spin (I = 7/2). The spectral was generated based on a spin Hamiltonian appropriate for these spectra previously reported in [52,53,54,55]. EPR Hamiltonian parameters, MO coefficients, the Fermi contact coefficient (*K*), and the dipolar hyperfine coupling parameter (P) were calculated using the LCAO-MO model developed by Kivelson and Lee [56] for vanadyl ions.

The EPR data obtained in the present study (Table 2A) indicate that g_‖_ < g_⊥_ < g_e_ (g_e_ = 2.0023) and A_‖_ > A_⊥_, revealing that V^4+^ ions incorporated in the phosphate network are present in the form of vanadyl ions (VO^2+^) [52,53,57] in a pyramidal site such as C_4v_ symmetry (an octahedral coordination with a tetragonal compression). The prevalent axial distortion of the VO^2+^ octahedral oxygen complex along the V = O bond is assumed to be the cause of relatively constant values for all the studied glasses, showing only a very slight variation with the content [58]. In [53], the value of A tensor was connected with the covalence degree between the paramagnetic ion and its ligands. The smaller the A value, the higher the covalence degree. The present results indicate a consistently higher A_‖_ value compared with A_⊥_ and therefore a great ionicity of the bonds in their axial direction. This statement is also supported by the values of MO coefficients (Table 2B), which show an ionic character for in-plane σ and π bonds (β_2_^2^ ~ 1) but a stronger covalency for out-of-plane vanadyl oxygen (ε_π_^2^ ~ 0.6), which is also consistent with the square-pyramidal coordination of vanadium ions in these glasses. *K* is a dimensionless Fermi contact interaction parameter [52] representing the amount of unpaired electron density at the vanadium nucleus location. The value obtained for the present glass system (k ~ 0.7) is in agreement with the literature [52,54,57,58,59], indicating a poor contribution of the vanadium 4s orbital to the vanadyl bond. The calculated dipolar hyperfine coupling parameter value (P ~ 142 cm^−1^) is also in agreement with the literature [26,52,54,57,58,59].

**Figure 6:**
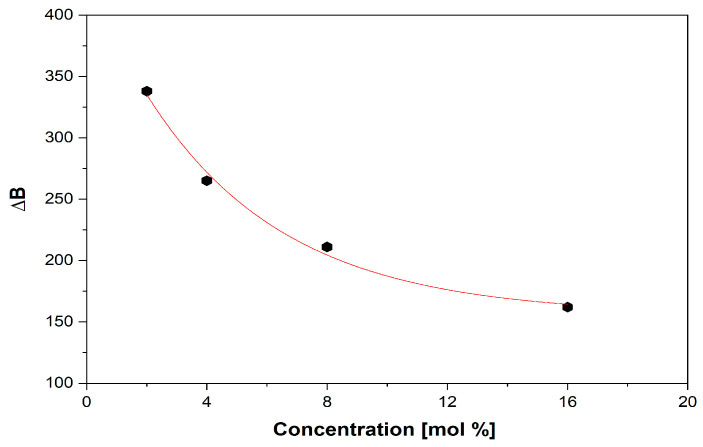
Decrease in the broad line (∆B) with increasing vanadium content.

With x < 4 mol% content of vanadium ions, the network is dominated by polymeric phosphate long-range structures with isolated paramagnetic ions in a specific position. As the content of vanadium ions increases (x ≥ 4 mol %), phosphate network depolymerization is fastened, and the isolated distribution of metallic ions is affected, allowing the appearance of dipole–dipole interactions between them. EPR data suggest the network-modifier role of V_2_O_5_ for x ≥ 4 mol %. For x > 4 mol %, the EPR spectra appear as a superposition of two signals, one with a well-resolved *hfs* structure typical of isolated ions and a second broad line typical of clustered ions. Figure 6 illustrates the decrease in the broad line with increasing vanadium concentration over 4 mol %, which correlates with previously reported data [54], indicating that superexchange magnetic interactions appear in V^4+^–O^2^–V^4+^ chains; this is consistent with the network-former role of vanadium oxide, which isolates the phosphate structures [26]. It was previously reported [48] that the presence of one fluorine ion may coordinate V^4+^ ion transposition with “yl” oxygen and substitute the 6th. This would lead to increased g‖ and A‖ values and a broadening of the vanadium *hfs* lines. This effect was not observed in the present glass system, although it contains fluorine ions.

#### 2.1.5. FT-IR

**Figure 7:**
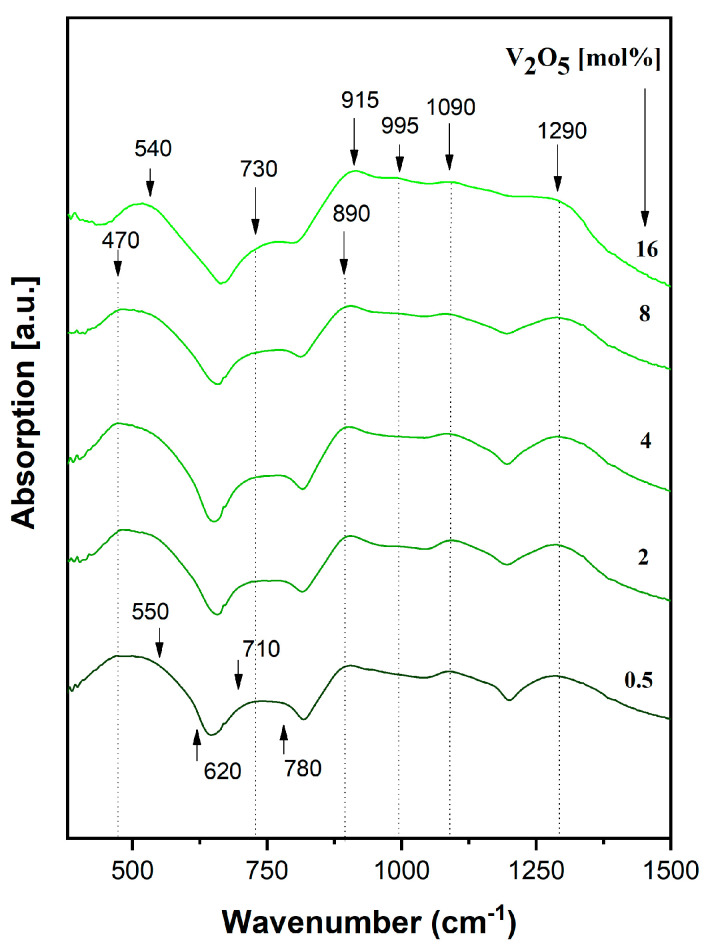
IR spectra of a xV_2_O_5_∙(100 − x) [CaF_2_∙3P_2_O_5_∙CaO] glass system.

IR spectra of the xV_2_O_5_∙(100 − x) [CaF_2_∙3P_2_O_5_∙CaO] glass system with 0 ≤ x ≤ 16 mol% in the range of 400–1400 cm^−1^ are presented in Figure 7. For the purposes of comparison and discussion, the relative intensities of the assigned vibrational-band IR spectra were normalized [60].

Phosphate networks traditionally indicate specific bands in the 450–1300 cm^−1^ [26,47,49,61,62,63,64], which are clearly visible in the present glass system starting with less concentrated vanadium (x = 0.5 mol %):

(a) Bands at ~ 475 cm^−1^, 550 cm^−1^, and 620 cm^−1^ are attributed to PO_2_ bending vibrations in P–O–P and O–P–O chains in PO_4_ groups and ring phosphate units;
(b) Bands at ~ 710 cm^−1^ and ~780 cm^−1^ are attributed to P–O–P symmetrical stretching vibrations in the ring phosphate units;
(c) Bands at ~ 890 cm^−1^ and ~ 915 cm^−1^ are assigned to asymmetrical stretching vibrations in the ring phosphate units;
(d) Bands at ~ 995 cm^−1^ and ~ 1090 cm^−1^ are attributed to PO_2_ stretching vibrations due to free oxygen atoms and a solid-state effect; and
(e) The band at ~1290 cm^−1^ belongs to the stretching vibrations of the phosphate network P = O double bond.

No significant changes were observed in the shape of IR spectra with up to 4 mol% vanadium content.

As vanadium content increases over 4 mol%, the band at 550 cm^−1^ increases in intensity and it is shifted to 540 cm^−1^ due to the superposition of phosphate bands and lattice vibrations of the V_2_O_5_ oxide network, especially at 16 mol % vanadium content. The bands at 620 and 710 cm^−1^ decrease in intensity until disappearance in the glasses with 16 mol %, due to the depolymerization effect of vanadium oxide. The band at 780 cm^−1^ is shifted towards 730 cm^−1^, mainly as a result of changes in the P–O–P length and a disorder effect produced by the depolymerization process induced by high vanadium content. The band at 890 cm^−1^ increases in intensity. Stretching vibrations in V–O bonds of VO_4_ tetrahedra are superpositioned over the stretching P–O–P vibration modes—an effect that is visible in the IR spectra over 4 mol %.

With a high content of vanadium introduced in the phosphate network (16 mol %), the bands at 915 cm^−1^, 995 cm^−1^, and 1090 cm^−1^ are better-located and shaped. The bands at 915 cm^−1^ and 995 cm^−1^ result from the superposition of P–O–P stretching vibration modes over V–O vibrations in VO_2_ trigonal by pyramids, and the band at 1090 cm^−1^ derives from the superposition of phosphate vibrations with V = O double-bond vibrations. The band at ~ 1290 cm^−1^ slowly shifts at 1300 cm^−1^ with high vanadium content and decreases in intensity due to V–O = P bonds, which weaken the double P = O bond in the process of network depolymerization [26,48,50,61,64,65,66,67].

Both EPR spectra and coefficients, as well as IR spectra, show the very clear vanadium oxide depolymerization effect.

### 2.2. In Vitro Biological Evaluation (Antitumoral Activity MTT Assay)

**Figure 8:**
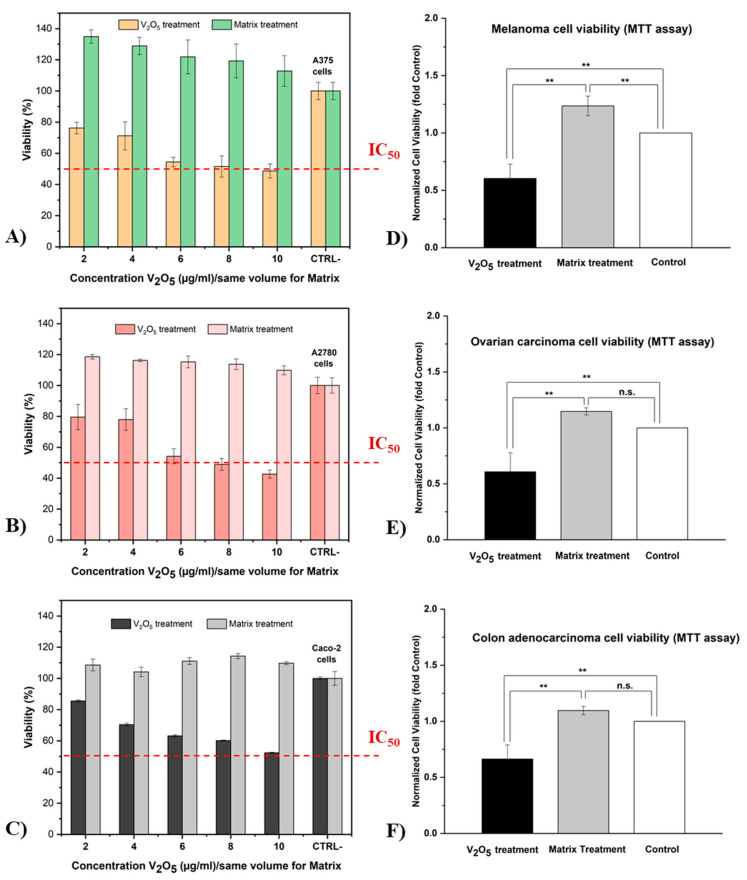
Percentage of viability of three different tumor cell lines (**A**, A375; **B**, A2780; and **C**, Caco-2) after 24 h incubation with glass extracts V7 and M in different concentrations (*n* = 5; error bars represent min–max values) andsignificant differences between the two treatments and the control (**D**,**E**) and within treatments (**F**) (mean ± SD, ** *p* < 0.01, n.s. = non-significant).

For in vitro biological evaluation, two glass samples (V7 and M) were selected from the xV_2_O_5_∙(100 − x) [CaF_2_∙3P_2_O_5_∙CaO] glass system with 0 ≤ x ≤ 16 mol%. The V7 glass sample (x = 16 mol% V_2_O_5_) was chosen due to its high solubility and the increased amount of doping. The M glass sample (x = 0 mol%) was chosen as the standard. The extracts were obtained as described in Section 3.3.3 and used as two treatments (Figure 8): (i) V_2_O_5_ treatment (V7 glass extract) and (ii) matrix treatment (M glass extract).

As shown in Figure 8A–C, an MTT assay was applied to several different human tumor lines (A375, A2780, and Caco-2) in order to highlight the antitumor character of vanadium ions from the V7 glass sample. Furthermore, dose–response experiments were performed for a limited concentration range (2, 4, 6, 8, and 10 μg/mL) with a relatively short incubation period (24 h) for both treatments. Cell viability without treatment/extract was chosen as the control (CTRL, 100%).

The results of the MTT assay showed that the V_2_O_5_ treatment exerted a strong antiproliferative effect on all tumor cell lines. This treatment caused a slight inhibition of tumor cell growth from the first concentration of 2 μg/mL V_2_O_5_. In addition, with increasing concentration, the efficacy of V_2_O_5_ treatment became increasingly evident, reaching a rate of inhibition of over 50% at the highest concentration (10 μg/mL V_2_O_5_) for the A375 and A2780 cell lines. According to ISO 10993-5 (in vitro cytotoxicity test), cell viability is determined as a percentage of the control; if cell viability is less than 70%, the material is cytotoxic [68]. In this case, concentrations of 6, 8, and 10 μg/mL V_2_O_5_ were considered to be cytotoxic for the studied tumor lines.

All types of cancer cells exhibit an increase in metabolic activity; therefore, redox homeostasis is of crucial importance for their survival. Disruption of this homeostasis with the purpose of triggering ROS-induced apoptotic signaling can be achieved with vanadium ions due to their increased redox potential. As demonstrated by Guerrero-Palomo et al. [29], this redox process is influenced by the oxidation state of the vanadium cation presented in the coordination sphere of the complex.

Concerning the matrix treatment (without vanadium), we can say that it did not interfere with cell viability and was therefore not cytotoxic for these tumor lines. Moreover, it provided an incentive physiological environment for cell attachment and therefore helped in cell development. Compared with V_2_O_5_ treatment and the CTRL-, it produced a considerable proliferative effect on melanoma, ovarian, and colon carcinoma cells. Additionally, as the concentration (dose) increased, the viability of tumor cells was slightly affected, although still not less than 100%. This effect can be explained by the composition of the matrix extract, which contains the main elements of glass M (trace elements such as Ca, P, and F). These elements are also naturally found in the human body and are biocompatible with biological systems [69].

According to statistical analyses (see Figure 8D–F), highly significant differences in cytotoxicity were found between the two types of treatment (*p* < 0.01). These differences were validated for all tested tumor cell lines. Surprisingly, no statistically significant differences were found between the matrix treatment and the control (cells grown without extract) for the A2780 and Caco-2 cell lines. However, significant differences were found between the matrix treatment and the control for the A375 line.

Therefore, our results suggest that the proliferation of the three tumor lines (A375, A2780, and Caco-2) was strongly affected by the applied V_2_O_5_-based treatment in a manner dependent on the concentration of V_2_O_5_ ions. Furthermore, the matrix treatment (without V_2_O_5_) provided an optimal environment for cell attachment and proliferation.

The incorporation of vanadium ions in various types of bioactive glasses and bioceramics is attractive for various biomedical applications, including drug delivery, tissue engineering, and cancer therapy. As shown throughout our work, V_2_O_5_-containing glass exerts in vitro strong cytotoxic effects against tumor cell lines A375, A2780, and Caco-2. However, owing to the lack of coherence of the chemical structure and the mechanism of action of vanadium ions, in vivo prediction is difficult to realize because experimental (chemical and biological) conditions are always more complex and significantly different from the conditions in clinical trials.

## 3. Materials and Methods

### 3.1. Preparation of Bioactive Glasses

The raw materials used in the present investigation, CaF_2_, P_2_O_5_, V_2_O_5_ (Alfa Aesar, Karlsruhe, Germany), and CaCO_3_ (Petr Švec—PENTA, Prague, Czech Republic), were of reagent-grade purity. The glass samples comprising xV_2_O_5_∙(100 − x) [CaF_2_∙3P_2_O_5_∙CaO] with 0 ≤ x ≤ 16 mol% were prepared by weighting suitable proportions of each component. The powders were mixed and melted in Al_2_O_3_ crucibles at 1170 °C in an electric furnace (PLF 110/6 Chamber Furnace) for 15 min. Homogenized melts were quenched to room temperature (RT) by pouring them on a stainless-steel plate and quickly pressing it with another plate. The prepared glass samples were mechanically (crushed) and chemically (dissolved) processed according to the analysis package used.

### 3.2. Sample Characterization

#### 3.2.1. Dissolution Test

We used dissolution tests in order to better understand the dynamics of doping the phosphate glasses. These tests were performed for all the powder samples under the same experimental conditions, deionized water as an immersion liquid at a temperature of 37 °C, which we chose to ensure a close resemblance to the temperature of the human body. The relative weight loss (*W_loss_*) of each sample after immersion was expressed as a percentage and calculated according to the following equation:

(2) $$W_{loss}=\frac{m_{i}-m_{f}}{m_{f}}\times 100$$

where *m_i_* is the initial weight of each sample, and *m_f_* is the weight after immersion in 10 mL deionized water.

For each sample, 100 mg was immersed in 10 mL of deionized water at 37 °C with continuous orbital shaking (300 rpm) on a titer plate shaker (Heidolph™ Titramax 1000, Hamburg, Germany) for 14 days. After incubation, the resulting suspensions were decanted and vacuum-filtered using Whatman-41 filter paper. The resulting sediment was then dried at RT for 3 days and weighted using a Precise XT220A analytical balance with a self-calibration system (SCS).

#### 3.2.2. pH Evolution

In order to assess pH changes over time, powdered glass (100 ± 0.5 mg) was immersed in 10 mL isotonic buffer (PBS, phosphate-buffered saline, pH 7.42) for up to 21 days at 37 °C. The PBS was synthesized according to AAT Bioquest, Inc. [70] (Table 3). The pH values were measured immediately after immersion for different incubation periods (1, 2, 5, 9, 14, and 21 days) using a digital pH meter (Titroline easy, Schott, Mainz, Germany).

The electrode was calibrated using standard pH values of 4.01, 7.00, and 10.01 before taking the pH measurements. Values were obtained with an estimated error of ± 0.02.

#### 3.2.3. SEM-EDS

To evaluate their composition and morphology, the glasses were analyzed using an SEM VEGA3 SBU-EasyProbe scanning electron microscope (Tescan, Bron, Czech Republic) with an energy-dispersive X-ray spectroscopy Quantax 200 EDS detector (Bruker, Berlin, Germany). The glass samples (powdered) were mounted on an aluminum stud using a double-sided adhesive carbon tape and measured in duplicate at RT. All quantitative EDS elementary data were calculated assuming the stoichiometry of the oxides and normalization to 100%.

#### 3.2.4. EPR

EPR measurements of powder samples were carried out at RT with a Bruker Biospin EMX spectrometer operating in the X band (9–10 GHz). The EPR parameters were set at 100 KHz modulation frequency, microwave power of 10 mW, modulation amplitude of 3 G, time constant of 2.56 ms, scan time of 61 s, and receiver gain of 103. To avoid the alteration of the glass structure due to ambient conditions, especially humidity, samples were powdered and enclosed in tubular holders of the same caliber. Equal quantities of samples were studied.

#### 3.2.5. FT-IR

Fourier transform infrared (FT-IR) absorption spectra were recorded using a JASCO 4100 spectrometer with a spectral range of 350 cm^−1^ to 4000 cm^−1^. Detection was carried out with a DLATGS detector with a KBr window at RT. The samples were prepared using the KBr pellet technique and measured at a resolution of 4 cm^−1^ with 256 scans/sample.

### 3.3. In Vitro Cytotoxicity Tests

#### 3.3.1. Cell Culture

Three cancer cell lines were studied: the A-375 (CRL-1619™) human melanoma cell line, the Caco-2 (HTB-37™) human colon adenocarcinoma cell line from the ATCC (American Type Culture Collection, Rockville, MD, USA), and the A2780 human ovarian carcinoma cell line from the ECACC (European Collection of Authenticated Cell Cultures, Salisbury, UK).

All cell lines were initially grown and then cultured using fresh medium supplemented as recommended by the suppliers; the A375 cell line was maintained in Dulbecco’s Modified Eagle Medium (DMEM) containing 4.5 g/L glucose 2 mM L-glutamine supplemented with 10% fetal bovine serum (FBS) and without antibiotics; the Caco-2 cell line was cultivated in Minimum Essential Medium (MEM), which contained 2 mM L-glutamine and % FBS without antibiotics; and the A2780 cell line was maintained in Roswell Park Memorial Institute (RPMI)–1640 medium supplemented with 2 mM L-glutamine, 10% FBS, 100 IU/mL penicillin, and 100 μg/mL streptomycin as antibiotics. Cells were plated in 25 cm^2^ tissue culture flasks and grown under standard conditions: 37 °C, 95% humidity, and 5% CO_2_ in air.

#### 3.3.2. Cell Subculture

To subculture the cells, they were divided, and the culture medium was replaced with fresh medium every 2–3 days as follows. First, the old medium was removed, and the cells were rinsed briefly with phosphate-buffered saline (PBS) at least 2 times. Five hundred milliliters of trypsin was then added, and the flask was incubated at 37 °C under 5% CO_2_ for 5–10 min. After the cells had detached from the lower part of the flask, 2 mL of fresh medium was added for trypsin inactivation. The slurry was then collected in a 15 mL tube and centrifuged at 800 g at RT for 10 min. After centrifugation, the supernatant was removed, and the pellet was mixed with 1 mL of fresh medium. The new culture was divided into two parts and transferred to two new flasks with 10 mL of fresh medium.

#### 3.3.3. Preparation of Extracts

Based on structural analyses and dissolution tests, two glass samples were chosen for in vitro bioactivity assessment (V7 and M). Each sample was extracted in deionized water (Milli-Q, Millipore GmbH, Schwalbach, Germany) with a concentration of 10 mg powder sample per 1 mL deionized water and incubated for 10 days at 37 °C with discontinuous stirring. After incubation, the formed colloidal solutions were centrifuged at 9000 rpm at RT for 10 min and filtered using a Whatman paper filter. Moreover, the obtained extracts were refiltered through a 0.22 μm Millex-GP syringe filter unit (Millipore, catalog number SLGP05010) and diluted with culture medium to obtain five serial concentrations of extract (2, 4, 6, 8, and 10 V_2_O_5_ μg/mL). For the matrix sample (without doping), we used the same volumetric quantities as those for extract V7.

#### 3.3.4. MTT Cell Proliferation Assay

The cells were grown until they were confluent. Then, they were trypsinized, and two hundred microliters of suspension containing 5 × 10^4^ cells was seeded in each well of a 96-well microtiter plate (Eppendorf, Germany) and incubated overnight at 37 °C with 5% CO_2_. On the following day, the medium was replaced, and the cells were treated with the two glass extracts (M and V7) in various concentrations (2, 4, 6, 8, and 10 µg/mL) and maintained at 37 °C with 5% CO_2_ for 24 h. After the incubation period, the old medium was removed from the wells and rinsed with 150 µL PBS (Ca- and Mg-enriched). Then, MTT (3-[4,5-dimethylthiazol-2-yl]-2,5 diphenyl tetrazolium bromide) was added, resulting in a final volume of 150 µL per well. The microtiter plate was then incubated again for 2 h at 37 °C with 5% CO_2_. After incubation, the MTT was removed, 150 µL of dimethyl sulfoxide (DMSO) was added over each well, and the cell viability was measured using a microplate reader (Synergy HT Multi-Mode BioTek, USA) at 550 and 630 nm (for background). The data are presented as the percentage of viable cells calculated according to Equation (2).

% Cell Viability = [(OD_s_ − OD_b_)/(OD_c_ − OD_b_)] × 100 (3)

where OD_s_is the optical density (in units) for the sample, OD_b_ is the optical density for the blank wells, and OD_c_ is the optical density for the control wells. The results are expressed as percent survival relative to an untreated control (CTRL-), and the experiment was performed with five repetitions for each treatment concentration to ensure a better and more accurate prediction.

#### 3.3.5. Statistical Analysis

All biological experiments were performed at least twice. Data are expressed as survival rate versus untreated control (CTRL-) for the cell viability of each treatment and mean ± standard deviation (SD) for each treatment type. For cell culture experiments, one replication represents the average of five wells containing glass-extract-treated or -untreated cells. In order to determine significant differences between types of treatments, a one-way analysis of variance (ANOVA) was performed, followed by Tukey’s HSD multiple range test. The significance of differences was defined at the 1% level (*p* < 0.01).

## 4. Conclusions

V_2_O_5_-doped phosphate glasses were successfully prepared by the conventional melt-quenching method with a chemical composition of xV_2_O_5_∙(100 − x) [CaF_2_∙3P_2_O_5_∙CaO] (where x = 0, 0.5, 1, 2, 4, 8, and 16 mol%) by varying the vanadium pentoxide content.

Dissolution tests demonstrates that the addition of V_2_O_5_ does not affect the dissolution rate of the prepared materials.

All glasses caused a decrease in the pH value of the PBS solution, proving the reactivity of the system. This phenomenon was caused by a rapid ion exchange between network-modifier ions (here, F^−^ and V_n_O_2n+1_) attached to non-bridging oxygen ions (NBOs) and HO from the solution.

SEM analysis illustrated that the structure and the homogeneity of vanadium-containing phosphate glasses do not change with doping concentration. EDS analysis confirmed the presence of all elements (P, O, Ca, and V) according to the nominal composition.

EPR revealed a well-resolved hyperfine structure (hfs) typical of vanadyl ions in a C_4v_ symmetry for glasses with 0.25 ≤ x ≤ 4 mol % V_2_O_5_. Additionally, with x > 4 mol% V_2_O_5_, the spectra showed the superposition of two EPR signals, one due to an *hfs* structure and the other consisting of a broad line typical of clustered ions.

FT-IR spectra of the xV_2_O_5_∙(100 − x) [CaF_2_∙3P_2_O_5_∙CaO] glass system showed the presence of all structural units characteristic of P_2_O_5_ in the glass structure, as well as a very clear depolymerization effect due to vanadium oxide (V_2_O_5_).

MTT assay showed that V_2_O_5_-based treatment has a strong antitumoral effect on cell growth, whereas matrix treatment does not interfere with cell viability and is therefore not cytotoxic for tested cell lines. In addition, the V_2_O_5_-based treatment has a visible inhibitory effect on tumor cells (from the lowest concentration used, i.e., 2 μg/mL V_2_O_5_) that increases directly proportionally to V_2_O_5_ concentration. Statistical analyses showed significant differences in cytotoxicity between the two types of treatment (*p* < 0.01).

In conclusion, the use of vanadium-containing bioglass particles should be encouraged for the development of new biomaterials and devices with biomedical applications, including innovative drug delivery methods and effective new cancer therapy options.

## Figures and Tables

**Table 1:** The chemical elements present in the surface of glass powders evaluated by EDS spectroscopy.

	Glass Code	Element Wt (%)			
No.		O	P	Ca	V
1	M	50.17	28.50	21.28	─
2	V1	47.95	30.84	20.37	0.83
3	V2	46.09	31.30	21.80	0.85
4	V3	44.74	30.38	24.05	0.83
5	V4	43.70	29.82	23.99	2.49
6	V5	42.59	33.32	18.68	5.40
7	V6	37.95	33.49	18.05	10.51
8	V7	34.17	30.29	16.25	19.29

**Table 2:** (**A**). EPR parameter and MO coefficients for a xV_2_O_5_∙ (100 − x) [CaF_2_∙3P_2_O_5_∙CaO] glass system with 0 ≤ x ≤ 4 mol%. (**B**)**.** MO coefficients for a xV_2_O_5_∙ (100 − x) [CaF_2_∙3P_2_O_5_∙CaO] glass system with 0 ≤ x ≤ 4 mol%.

**(A)**				
**x (mol% V2O5)**	**g** **_⊥_**	**g_‖_**	**A** **_⊥_** **(10^−4^ cm^−1^)**	**A_‖_** **(10^−4^ cm^−1^)**
0.25	1.99	1.93	69	184
0.5	1.99	1.93	64	185
1	1.99	1.92	55	176
2	1.99	1.93	63	185
4	1.99	1.92	59	184
**(B)**				
**x (mol% V2O5)**	**k**	**P (10^−4^ cm^−1^)**	**β_2_^2^**	**ε** **_π_^2^**
0.25	0.79	136	0.9	0.6
0.5	0.73	142	1.1	0.5
1	0.71	138	0.9	0.6
2	0.73	142	1.1	0.5
4	0.70	146	0.9	0.6

**Table 3:** The reagents used in the preparation of the 1 L buffer solution (PBS, pH 7.4).

Reagent Chemicals (Chempur)	Molecular Weight (g/mol)	Amount (mg)	Concentration (M)
NaCl	58.4	8000	0.137
KCl	74.551	200	0.0027
Na_2_HPO_4_	141.96	1440	0.01
KH_2_PO_4_	136.086	245	0.0018

## Data Availability

Not applicable.
